# Is mRNA decapping by ApaH like phosphatases present in eukaryotes beyond the Kinetoplastida?

**DOI:** 10.1186/s12862-021-01858-x

**Published:** 2021-06-23

**Authors:** Paula Andrea Castañeda Londoño, Nicole Banholzer, Bridget Bannermann, Susanne Kramer

**Affiliations:** 1 Zell- Und Entwicklungsbiologie, Biozentrum, Universität Würzburg, Würzburg, Germany; 2 Department of Medicine, University of Cambridge, Cambridge, UK

**Keywords:** ApaH like phosphatase, ApaH, ALPH, *Trypanosoma brucei*, mRNA decapping, m7G cap, mRNA cap, ALPH1, Kinetoplastida

## Abstract

**Background:**

ApaH like phosphatases (ALPHs) originate from the bacterial ApaH protein and have been identified in all eukaryotic super-groups. Only two of these proteins have been functionally characterised. We have shown that the ApaH like phosphatase ALPH1 from the Kinetoplastid *Trypanosoma brucei* is the mRNA decapping enzyme of the parasite. In eukaryotes, Dcp2 is the major mRNA decapping enzyme and mRNA decapping by ALPHs is unprecedented, but the bacterial ApaH protein was recently found decapping non-conventional caps of bacterial mRNAs. These findings prompted us to explore whether mRNA decapping by ALPHs is restricted to Kinetoplastida or could be more widespread among eukaryotes.

**Results:**

We screened 827 eukaryotic proteomes with a newly developed Python-based algorithm for the presence of ALPHs and used the data to characterize the phylogenetic distribution, conserved features, additional domains and predicted intracellular localisation of this protein family. For most organisms, we found ALPH proteins to be either absent (495/827 organisms) or to have non-cytoplasmic localisation predictions (73% of all ALPHs), excluding a function in mRNA decapping. Although, non-cytoplasmic ALPH proteins had in vitro mRNA decapping activity. Only 71 non-Kinetoplastida have ALPH proteins with predicted cytoplasmic localisations. However, in contrast to Kinetoplastida, these organisms also possess a homologue of Dcp2 and in contrast to ALPH1 of Kinetoplastida, these ALPH proteins are very short and consist of the catalytic domain only.

**Conclusions:**

ALPH was present in the last common ancestor of eukaryotes, but most eukaryotes have either lost the enzyme, or use it exclusively outside the cytoplasm. The acceptance of mRNA as a substrate indicates that ALPHs, like bacterial ApaH, have a wide substrate range: the need to protect mRNAs from unregulated degradation is one possible explanation for the selection against the presence of cytoplasmic ALPH proteins in most eukaryotes. Kinetoplastida succeeded to exploit ALPH as their only or major mRNA decapping enzyme. 71 eukaryotic organisms outside the Kinetoplastid lineage have short ALPH proteins with cytoplasmic localisation predictions: whether these proteins are used as decapping enzymes in addition to Dcp2 or else have adapted to not accept mRNAs as a substrate, remains to be explored.

**Supplementary Information:**

The online version contains supplementary material available at 10.1186/s12862-021-01858-x.

## Background

Eukaryotic phosphatases play essential roles in regulating many cellular processes and can be classified in several ways, based on catalytic mechanism, substrate specificity, ion requirements and structure. One four-group classification distinguishes phosphoprotein phosphatases (PPPs), metal-dependent protein phosphatases (PPM, sometimes classified as a subgroup of PPP), protein tyrosine phosphatases and aspartic acid-based phosphatases [1]. The eukaryotic PPP group includes the Ser/Thr phosphatases PP1, PP2A, PP2B, PP4, PP5, PP6 and PP7 [1, 2] but has been extended to include three families of bacterial origin: Shewanella-like SLP phosphatases, Rhizobiales-like (RLPH) phosphatases and ApaH-like (ALPH) phosphatases [3–6].

ApaH like phosphatases (ALPHs) evolved from the bacterial ApaH protein and were present in the last common ancestor of eukaryotes [3, 5]. They are widespread throughout the entire eukaryotic domain, although they have been lost in certain sub-branches such as land plants and chordates [6]. To the best of our knowledge, only two ALPHs have been functionally characterised to date. One is the *S. cerevisiae* ALPH protein (YNL217W), a Zn^2+^ dependent endopolyphosphatase of the vacuolar lumen [7] that is also active with Co^2+^ and possibly Mg^2+^ [8]. The enzyme’s main function is the cleavage of vacuolar poly(P). A function of Ppn2 in stress response is suggested by the fact that the increase in short polyphosphate upon Ppn2 overexpression correlates with an increased resistance to peroxide and alkali [9]. The second characterised ALPH protein is ALPH1 of the Kinetoplastida *Trypanosoma brucei*: we recently found that ALPH1 is the only or major trypanosome mRNA decapping enzyme [10], the enzyme that removes the m^7^ methylguanosine (m^7^G) cap present at the 5´end of most eukaryotic mRNAs. This finding was surprising, as all other known eukaryotic mRNA decapping enzymes belong to a different enzyme family, the nudix hydrolases (the prototype is Dcp2). Trypanosomes lack orthologues to Dcp2 and all decapping enhancers associated with Dcp2, but use the ApaH like phosphatase ALPH1 instead. *T. brucei* ALPH1 consists of the catalytic domain with N- and C- terminal extensions of about equal sizes, that appear unique to Kinetoplastida and bear no similarities to any known domains.

Interestingly, recent data indicate, that the bacterial precursor protein of ALPH, ApaH, may have an analogous function to Trypanosome ALPH1 in decapping of bacterial mRNAs. In vitro, ApaH cleaves diadenosine tetraphosphate (Ap4A) into two molecules of ADP [11, 12], but is also active towards other NpnN nucleotides (with n ≥ 3) [13–15]. Deletion of the ApaH gene causes a marked increase in Np4A levels and a wide range of phenotypes [16–23]. Np4A was therefore suggested to act as an alarmone (a signalling molecule involved in stress response), but no Np4A receptor has yet been identified. Instead, recent data show that stress induced increase in Np4A levels cause massive nucleoside-tetraphosphate capping of bacterial mRNAs [24] mostly or entirely caused by usage of Np4A as non-canonical transcription initiation nucleotide [25]. Many other dinucleoside polyphosphates can be used for co-transcriptional capping even in the absence of stress, including methylated versions [26]. ApaH is the major decapping enzyme for all dinucleoside polyphosphate caps [24, 26], suggesting that its main function is to regulate gene expression by modulating the mRNA decapping process. Intriguingly, the enzyme can both enhance decapping (by decapping nucleoside-tetraphosphate capped RNA) and inhibit decapping (by cleaving Np4A and preventing its incorporation to the mRNA).

Puzzled by these novel functions of bacterial ApaH and trypanosome ALPH1 in mRNA decapping, we here set out to investigate whether mRNA decapping is a major function of eukaryotic ALPHs, or whether this function is restricted to trypanosomes. We developed a Python algorithm for the identification of ALPHs in all available eukaryotic reference proteomes and identified 441 ALPHs in 332/827 proteomes. We show that most ALPH proteins consist exclusively of the catalytic domain and many have predicted transmembrane regions or signal peptides and predicted non-cytoplasmic localisation, indicating functions distinct from mRNA decapping. We show in vitro mRNA decapping activity for three of these non-cytoplasmic ALPH proteins, indicating that, similar to bacterial ApaH, eukaryotic ALPHs accept non-physiological substrates and thus are likely to have a rather wide substrate range. The data indicate that there may be selection against the presence of cytoplasmic ALPH proteins in eukaryotes, possibly to protect mRNAs from unregulated degradation. Apart from the kinetoplastids, only 71 organisms possess ALPH proteins with cytoplasmic localisation predictions. In contrast to the Kinetoplastida, these ALPH proteins consisted (with 2 exceptions) of the catalytic domain only and all organisms have an orthologue to Dcp2 (with 3 exceptions likely caused by genome incompleteness).

## Results

### Identification of ApaH like phosphatases in available eukaryotic proteomes

ALPHs belong to the PPP family of phosphatases and possess the four conserved signature motifs (motif 1–4) of this family, GDxHG, GDxxDRG, GNHE, and HGG, sometimes with conservative substitutions [2]. One distinctive feature of ALPHs are two changes in the GDxxDRG motif: The second Asp is replaced by a neutral amino acid and the Arg residue is replaced by Lys. In addition, ALPHs have two C-terminal motifs [3, 6] that we here call motif 5 and 6. We screened 827 complete ukaryotic proteomes (Additional file 3: Table S1a) for the presence of ApaH like phosphatases with a home-made Python algorithm; these included all reference proteomes available on UniProt [27] and all available Kinetoplastida proteomes available on TriTrypDB [28, 29]. The algorithm is based on recognising the six sequence-motifs that are characteristic for ALPHs. The matrices used to define these motifs were optimised stepwise using yeast and Kinetoplastida proteomes to not miss any ALPH (controlled by BLAST) while on the other hand not to recognise PPPs, and in particular not the closely related phosphatases SLP, RLPH and ApaH (sequences taken from [6]). The final algorithm also included restrictions on distances between the motifs 1 to 2, 2 to 3 and 5 to 6 that we found highly conserved. More details are in material and methods. BLAST screens on selected proteomes without ALPH proteins revealed that ALPHs were either fully absent or, in rare cases, present in a truncated version and missing at least one of the motifs, mostly the N- or C-terminal one. ALPH proteins with missing N- or C-terminal motifs may have arisen from sequencing or annotation errors and were not included in the final list (Additional file 4: Table S2c) (11 proteins in total, all belong to phylogenetic groups with ALPH proteins present). Only for the Kinetoplastida, ALPHs with wrongly annotated start codons were manually included (based on comparison with related Kinetoplastida and available genome information).

**Fig. 1:**
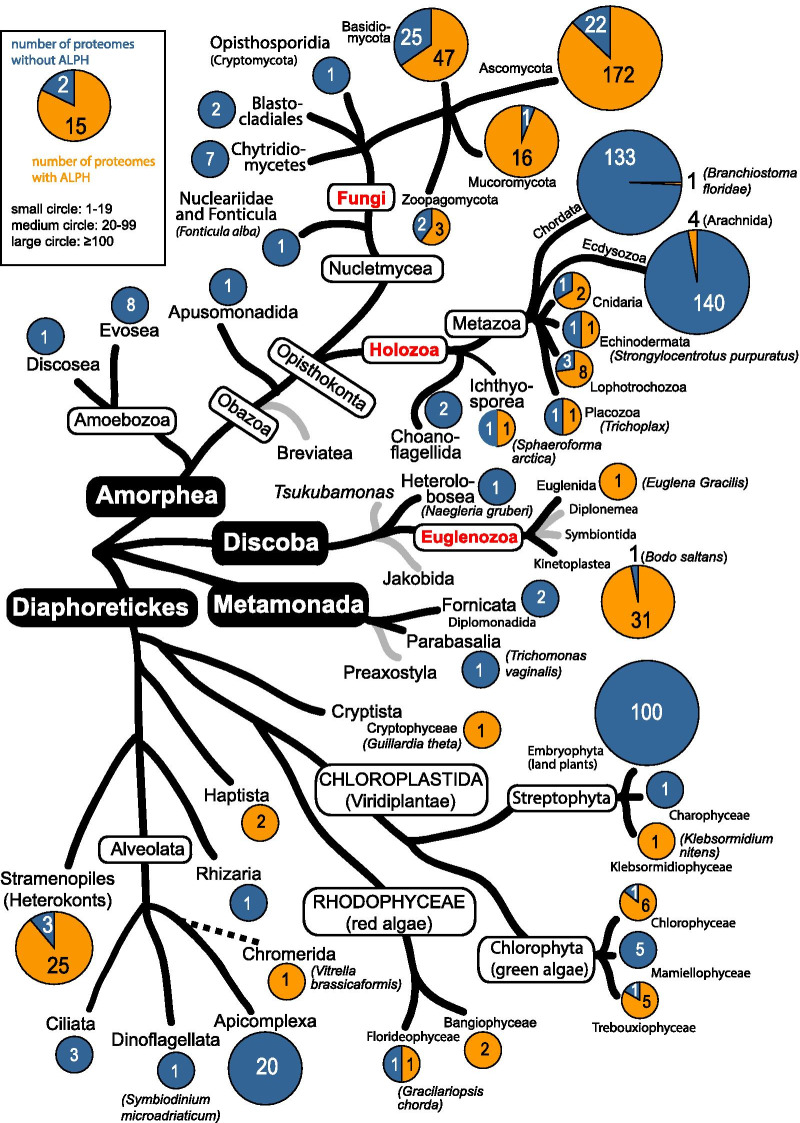
Presence and absence of ALPH in the different eukaryotic subgroups. 827 eukaryotic proteomes were screened for the presence of ALPH. Absence or presence of ALPH is shown in pie diagrams in blue and orange, respectively, for each phylogenetic group. The diameter of a circle roughly reflects the number of available proteomes. The phylogenetic tree was taken from [30]. All organisms are listed in Additional file 3: Table S1

Figure 1 summarises all organisms of this study in phylogenetic groups based on the latest eukaryotic classification suggested by [30]. For each group, the fraction of organisms with and without ALPH is indicated in orange and blue, respectively. 332 of all 827 organisms included in this study have at least one ALPH, and these organisms are distributed in a patchy way throughout all eukaryotes. Most Euglenozoa have ALPHs (32 of 33), many Stramenopiles (25 of 28) and Fungi (238 of 298), but also Rhodophyceae (3 of 4), Chlorophyta (11 of 18), Haptista (2 of 2) and some Metazoa (17 of 296). ALPHs are absent from land plants (100 proteomes tested), Apicomplexa (20 proteomes tested), Ciliata (3 proteomes tested) and Dinoflagellates (1 proteome tested). They are largely absent from Chordata (134 proteomes tested, only *Branchiostoma floridae* has ALPH) and from Ecdysozoa (144 proteomes tested, only 4 *Arachnida* have ALPH) and fully absent from the few available proteomes of Amoebozoa (9 proteomes tested) and Metamonada (3 proteomes tested). A phylogenetic tree built from the catalytic domains of ALPHs mostly reflects the eukaryotic tree (Additional file 1: Figure S1). Taken together, the data indicate that ALPH was present in the last common ancestor of all eukaryotes and was then selectively lost in certain sub-branches. Our data fully agree with the data of [6], regarding absences and presences of ALPH proteins in eukaryotic subgroups. We extend the available dataset from 52 ALPHs (38 organisms) to 441 ALPHs (332 organisms), this way providing a better resolution of ALPH distribution across eukaryotes.

The aim of this work was not to analyse horizontal gene-transfer between eukaryotes, bacteria and archaea; however, we could confirm the presence of ALPHs in a subgroup (11/25) of *Myxococcales*, as described in [6] (Additional file 5: Table S3A). We detected ALPH in 1 of 285 archaean proteomes (OX = 1906665 GN = EON65_52185, UniProt: UP000292173) (Additional file 5: Table S3B); it is possible that this protein is a contamination. All prokaryotic ALPHs consisted mostly of the catalytic domain with almost no N- or C terminal extensions.

### General features of ApaH-like phosphatases

**Fig. 2:**
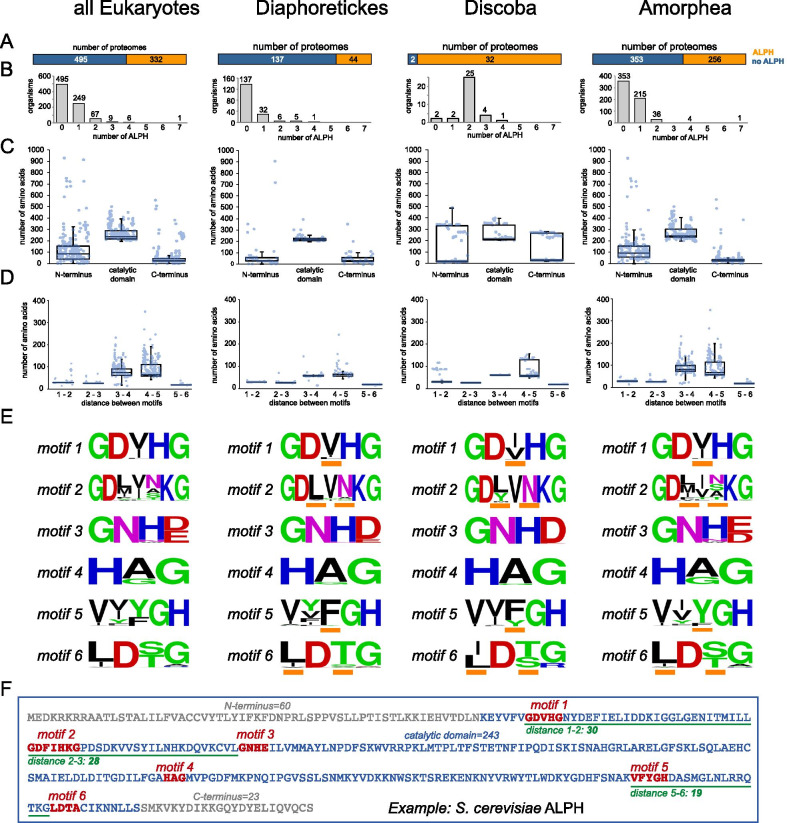
General Features of ALPHs. **A** Proteomes with (orange) and without (blue) ALPHs. **B** Number of ALPH proteins per organism. **C** Sizes of the different ALPH ‘domains’ (N-terminus, catalytic domain, C-terminus) are presented as box plot (waist is median; box is IQR; whiskers are ± 1.5 IQR) and in addition as individual data points (transparent blue circles). The catalytic domain is defined as the range between the first and last motif, with an additional six N-terminal amino acids and an additional eight C-terminal amino acids (compare **F**). **D** Distances between the six different motifs (in amino acids) are presented as box plot (waist is median; box is IQR; whiskers are ± 1.5 IQR) and in addition as individual data points (transparent blue circles). **E** Sequence motifs were created with WebLogo [31]. The most obvious differences between the three groups are marked with orange bars. **F** The sequence of ALPH from *S. cerevisiae* is shown as a typical example, to illustrate the position of the catalytic domain (blue), the N- and C-termini (grey) as well as all six motifs (red) and the distances between the motifs that we found most conserved (green). Details on all data presented in Fig. 2 can be found in Additional file 3: Table S1

The dataset was used to refine the characteristics of ALPH proteins (Fig. 2). 40% of all analysed organisms have at least one ALPH isoform. The highest percentage is found in Discoba with 94%, the lowest in Diaphoretickes with 24% (Fig. 2A). Of the 332 APLH-positive organisms, 25% have more than one ALPH isoform: most (81%) have two and with one exception no organism has more than four (Fig. 2B). Organisms with multiple ALPHs were mostly enriched among the Discoba (94% of organisms with ALPH had at least 2 ALPHs) (Fig. 2B). The vast majority of all ALPH proteins are very short and consist mainly of the catalytic domain with short N- and C-terminal extensions (Fig. 2C). The median size of the C-terminal extensions is 26 amino acids: only 52 of all 441 ALPH proteins have C-termini extending 100 amino acids and most of these (31) are ALPHs of Discoba. The size of the N-terminus is slightly more variable and has a median of 87 amino acids. Only 61 of all 441 ALPH proteins have N-termini extending 200 amino acids and many of these (28) belong to ALPHs of Discoba. The largest variance in the size of ALPH N- and C-termini is found in Discoba, reflecting the presence of two different ALPH variants in the Kinetoplastida (discussed below).

The amino acid distances between some of the ALPH motifs are highly conserved (Fig. 2D): 93.7% of all ALPHs have between 28 and 30 amino acids between motif 1 and 2 (72% have exactly 29). The distance between motif 2 and 3 is exactly 26 amino acids for 83.0% of ALPHs and between 25 and 33 for 98.0% of ALPHs. The distance between the two C-terminal motifs 5 and 6 is exactly 19 amino acids for 92.5% of ALPHs. The distances between motif 3 and 4 are well conserved in ALPHs of Diaphoretickes and Discoba (94% are between 55 and 64 amino acids) but less well within Amorphea. The distances between motif 4 and 5 are poorly conserved. Sequence motifs were created for all six motifs (Fig. 2E) [31]. Mostly, these are conserved with some group-specific preferences at certain positions indicated with orange bars (Fig. 2E). The ALPH sequence of *S. cerevisiae* is shown as an example, to illustrate the definitions of all features discussed above (Fig. 2F).

### ALPHs of Opisthokonts (fungi and holozoa)

**Fig. 3:**
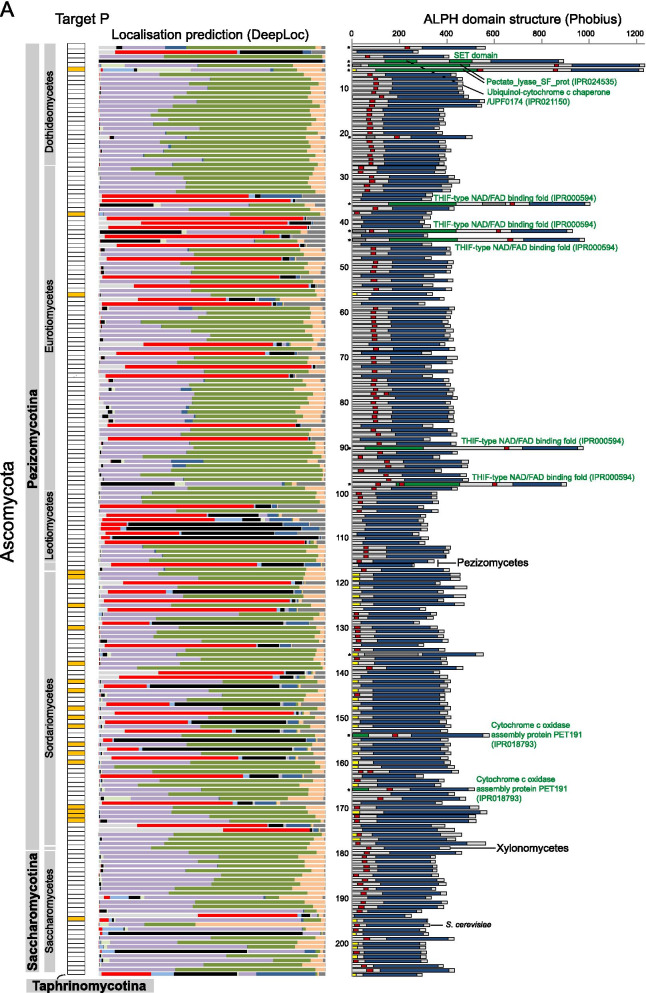

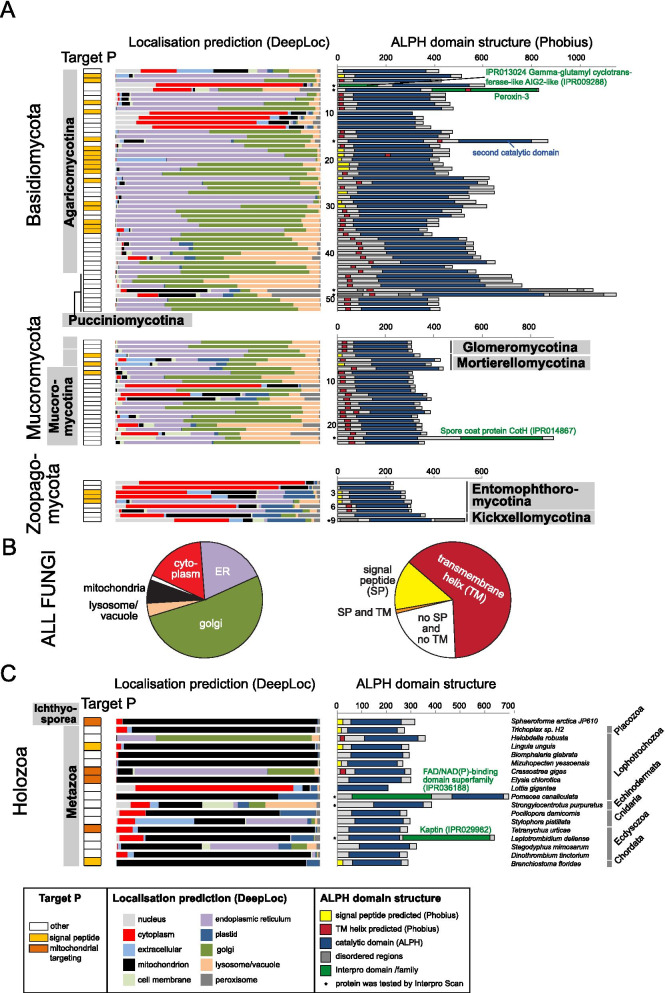
ALPHs of Opisthokonts. **A** ALPH proteins of Fungi are presented as schematics with their catalytic domain and additional predicted features (further domains, transmembrane helices, signal peptides) (right). Localisation predictions (Target P, DeepLoc) are shown on the left. The organism names and more details can be found in Additional file 3: Table S1b. **B** Pie charts summarizing localisation predictions (DeepLoc) (left) and predicted transmembrane helix or signal peptides (right) for ALPH proteins from all Fungi. **C** ALPH proteins from Holozoa are presented as in (**A**). More details are listed in Additional file 3: Table S1c

The majority of available ALPH sequences (292) are from Fungi, because the number of available proteomes is high (298) and 80% of these proteomes contain at least one ALPH. We investigated these ALPH sequences further by looking for predicted domains (Interpro [32]), signal peptides and trans-membrane helices (Phobius [33] and Target P [34]) and predicted localisation (DeepLoc [35] and WoLF PSORT [36]) (Fig. 3A and Additional file 3: Table S1b). ALPHs of Fungi have very short C-termini (median = 26 amino acids) and only slightly larger N-termini (median = 97 amino acids). Most fungal ALPHs (95.2%) do not contain any predictable domain in addition to the catalytic ALPH domain. Of the 14 ALPHs that have a further domain, three have domains with functions in cytochrome c complex assembly (IPR021150, IPR018793) indicating mitochondrial functions and five ALPHs have a THIF-type NAD/FAD binding fold, usually found in the ubiquitin activating E1 family, indicating a possible function in protein degradation. The ALPH of *Lentinula edodes* has a Peroxin-3 domain, indicating a peroxisomal function. Two ALPHs of *Rachicladosporium* have Pectate lyase domains, indicating a possible function in degradation of cell wall material. ALPH of *Phycomyces blakesleeanus* has a spore coat protein CotH (IPR014867) domain, a domain of bacterial origin with unknown function in eukaryotes. The ALPH of *Rhizopogon vesiculosus* has a second ALPH domain.

The most interesting finding was the presence of predicted trans-membrane regions and/or signal peptides within the C-terminal region in 78.1% of all fungal ALPH proteins: 184 ALPH proteins have predicted membrane helices (mostly one), a further 41 ALPH proteins have predicted signal peptides, 3 ALPH proteins have both predicted and only 64 ALPH proteins have neither predicted (Fig. 3A and B, Additional file 3: Table S1b). In agreement with these data, only 49 ALPH proteins (16.8%) have predicted cytoplasmic localisation (mostly the ones without predicted membrane helices and signal peptides) (Fig. 3A and B, Additional file 3: Table S1b). The remaining proteins are predicted to be in the golgi (52.1%), endoplasmatic reticulum (19.5%), mitochondrion (6.5%), lysosome/vacuole (3.8%), nucleus (2 proteins, 0.7%), peroxisome (1 protein, 0.3%) and extracellular (1 protein, 0.3%). Prediction data need to be considered with care in the absence of experimental evidence, but taken together, the data provide strong evidence for the majority of fungal APLH proteins being non-cytoplasmic. Experimental data confirm non-cytoplasmic localisation for the ALPH protein of *S. cerevisiae* (YNL217w, Ppn2): two high-throughput studies indicate vacuolar localisation [37, 38] and recent data show that Ppn2 is delivered to the vacuolar lumen via the multivesicular body pathway, where it functions as an endopolyphosphatase [7].

ALPHs are underrepresented in Holozoans (Fig. 1). In particular, all 130 vertebrate proteomes lack ALPHs and of the three available non-vertebrate proteomes of Chordata, only the Lancelet *Branchiostoma floridae* is ALPH-positive. Out of the 140 available Ecdysozoan proteomes, only four organisms (Arachnida species) contain ALPH. ALPHs are present in subgroups of Cnidarians (2/3), Echinodermatans (1/2), Lophotrochozoens (8/11), Placozoans (1/2) and Ichthyosporeans (1/2). 7 of these 18 Holozoan ALPH proteins have a predicted signal peptide or transmembrane helix at their C-termini and with the exception of ALPH from *Lottia gigantea*, all proteins have non-cytoplasmic localisation predictions, mostly to the mitochondria (13/18) (Fig. 3C, Additional file 3: Table S1c). Two proteins have an additional domain: ALPH of *Pomacea canaliculata* has a domain of the FAD/NAD(P)-binding superfamily N- terminal of the catalytic domain and ALPH of *Leptotrombidium deliense* may be interacting with actin, as it has a Kaptin domain C-terminal of its ALPH domain.

All the 42 organisms containing an ALPH protein with a cytoplasmic localisation prediction also have a Dcp2 homologue (Blast, Additional file 3: Table S1b and c), indicating that a function of the ALPH protein in mRNA decapping, if present, is not exclusive.

### ALPHs of diaphoretickes

**Fig. 4:**
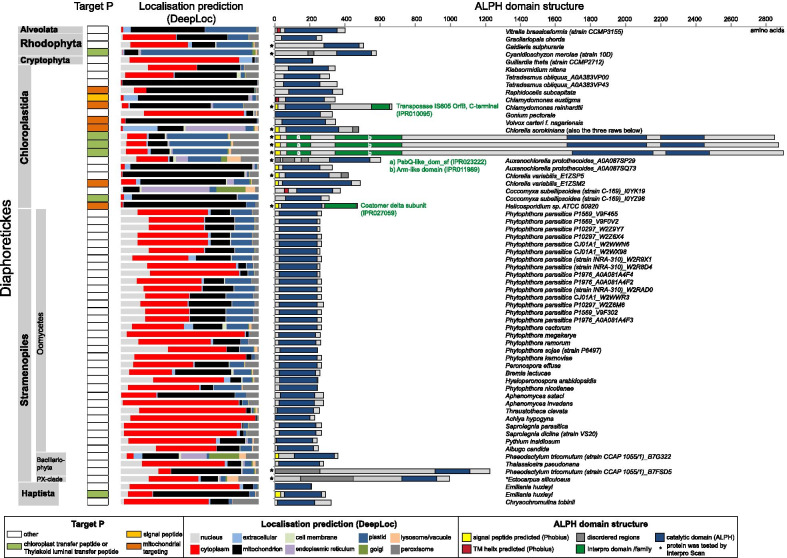
ALPHs of Diaphoretickes. ALPHs of Diaphoretickes are presented as schematics with their catalytic domain (dark blue) and additional predicted features (further domains, transmembrane helices and signal peptides) (right) and localisation predictions (Target P and DeepLoc) (left). More details are listed in Additional file 3: Table S1d

We found no ALPHs in land plants (100 proteomes), Apicomplexa (20 proteomes), Ciliata (3 proteomes) or Dinoflagellata (1 proteome). ALPH is present in 3 of 4 species of red algae, 11 of 18 species of green algae (Chlorophyta), in the filamentous green algae *Klebsormidium nitens*, in the photosynthetic Alveolate *Vitrella brassicaformis,* in the cryptophyte algae *Guillardia theta* and in the two available Haptista proteomes. 25 of 28 Stramenopiles have ALPHs: These are mostly (non-photosynthetic) Oomycetes, including for example *Phytophthora parasitica*: all the strains of this plant pathogen have three ALPH isoforms. Predicted signal peptides or transmembrane helices are present at the C-termini of many Chloroplastida ALPHs (11/19), as well as in ALPH proteins of the Alveolata *Vitrella brassicaformis,* the diatom *Phaeodactylum tricornutum* and the Haptista *Emiliania huxleyi;* in all cases the presence of transmembrane helices or signal peptides correlates with a predicted non-cytoplasmic localisation (Fig. 4). With two exceptions, all 19 ALPH proteins from Chloroplastida have non-cytoplasmic localisation predictions (11 mitochondrion, 4 chloroplast, 2 endoplasmic reticulum). In contrast, the remaining 74 ALPH proteins from non-Chloroplastida have mostly cytoplasmic localisation predictions, with only 7 exceptions (6 mitochondrion, 1 chloroplast).

The majority of ALPH proteins from Diaphoretickes is very short and, in particular ALPHs of Oomycetes, consist almost exclusively of the catalytic domain with very short N- and C-terminal extensions. Of all 63 Diaphoretickes ALPH proteins, only five have additional domains: the ALPH of *Chlamydomonas reinhardtii* has a predicted Transposase IS605 domain (a transposon of bacterial origin), the ALPH of *Helicosporidium* has a coatomer delta subunit domain and the three large and almost identical ALPH proteins of *Chlorella sorokiniana* have a PsbQ-like domain (IPR023222), an Arm-like repeat and a second ALPH domain. A PsbQ-like domain is typical for proteins of the photosystem II, consistent with localisation predictions of the three *Chlorella sorokiniana* ALPH proteins to the chloroplast.

Of the 29 Diaphoretickes that have ALPH proteins with predicted cytoplasmic localisation, 26 have a readily identifiable homologue to Dcp2 of *Arabidopsis thaliana* (in some cases only as a fragment as proteomes were not complete) (Additional file 3: Table S1d). The three remaining organisms with no identifiable Dcp2 homologue are *Chrysochromulina tobinii*, *Hyaloperonospora arabidopsidis* and *Pythium insidiosum*. However, closely related species to all three organisms have Dcp2 and it is likely that the absence of a Dcp2 is caused by genome incompleteness: the proteome of *C. tobinii* is 37% incomplete and the proteomes of *H. arabidopsidis* and *P. insidiosum* are 7% and 1% incomplete, respectively. Thus, any function of an ALPH protein in mRNA decapping is likely not exclusive but occurs in addition to Dcp2.

### ALPHs of Euglenozoa

ALPHs are present in all Kinetoplastida and in their close relative *Euglena gracilis* (Additional file 3: Table S1e). The only exception is the free-living, non-parasitic *Bodo saltans*.

**Fig. 5:**
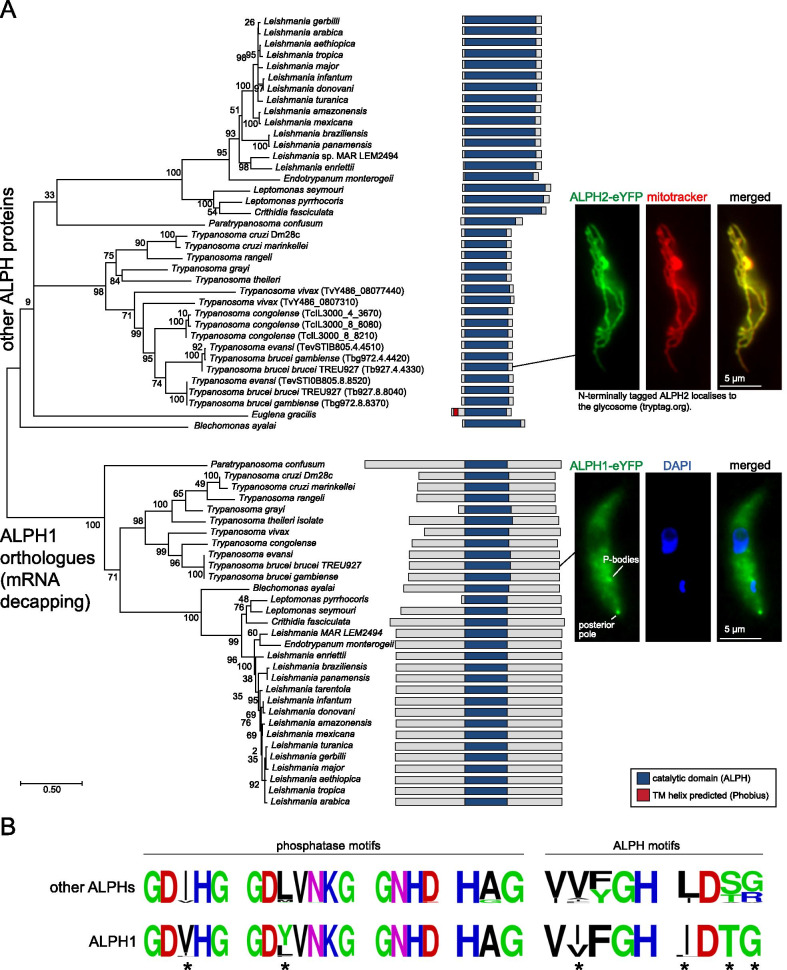
ALPHs of *Euglenozoa*. **A** ALPH proteins of *Euglenozoa* are presented schematically along a maximum likelihood phylogenetic tree based on an alignment of the catalytic domains. All details on *Euglenozoa* ALPHs are listed in Table S1e. The localisation of *T. brucei* ALPH1 and ALPH2 was determined by expressing eYFP fusion proteins. One representative fluorescent image is shown for each protein. At least three different clonal cell lines showed identical localisation. **B** Sequence motifs of the ALPH1 orthologues and the non-ALPH1 orthologues. Major differences are marked by asterisks

ALPHs of Kinetoplastida fall into two groups (Fig. 5A): each organism has exactly one ALPH that is homologous to *T. brucei* ALPH1, the mRNA decapping enzyme [10]. These ALPHs all have a C-terminal extension of between 220 and 278 amino acids and, with two exceptions (*Leptomonas pyrrhocoris* and *T. grayi*), they all have N-terminal extensions of a similar size. The in vitro mRNA decapping activity of *T. brucei* ALPH1 does not require the N-terminus [10], and the two ALPHs that lack the N-terminus are therefore also likely active in mRNA decapping. The fact that no Kinetoplastida strain has lost its ALPH1 homologue, and the absence of homologues to the canonical mRNA decapping enzymes (DCP1/DCP2), indicates that all Kinetoplastida rely on ALPH1 for mRNA decapping.

In addition to the ALPH1 homologue, all but one Kinetoplastid have between one and three additional ALPH proteins that consist exclusively of the catalytic domain (Fig. 5A). These ALPH proteins of *Leishmania*, *Leptomonas*, *Crithidia* and *Endotrypanum* have extensions within the catalytic domain that are mainly caused by enlarged distances between motif 1 and 2 (up to 115 nucleotides in *Leptomonas* and *Crithidia*, more than in any other ALPH) and between motif 4 and 5 (up to 156 nucleotides). The only Kinetoplastida that has no ALPH apart from ALPH1 is *Leishmania tarentolae*; blast searches identified a protein at the expected position in the genome which lacked both motif 5 and motif 6, indicating a recent loss of the protein.

Differences between the two different groups of Kinetoplastida ALPH proteins are also obvious within the phosphatase and ALPH motifs: several positions show major differences; most pronounced is the difference in motif 1 (GDVHG/GDIHG for ALPH1/non-ALPH1) and in motif 6 (IDTG/LDSG for ALPH1/non-ALPH1) (Fig. 5B). When a phylogenetic tree is constructed from the sequences of the catalytic domains, the Kinetoplastida ALPH1 homologues form a separate group, distant to the group of the non-ALPH1 homologues, indicating that the ALPH1 decapping enzyme has evolved only once in the last common ancestor of the Kinetoplastida (Fig. 5A).

Protein localisation predictions are difficult in Euglenozoa as these organisms have diverse and poorly defined targeting signals: localisation predictions from Phobius, DeepLoc and Target P are listed in Additional file 3: Table S1e but do not correlate well with each other, and, in particular not with our experimental data. The decapping enzyme *T. brucei* ALPH1, fused to eYFP either C- or N- terminally, localises to the cytoplasm, P-bodies and to the posterior pole of the cell and this cytoplasmic localisation is consistent with the essential function of ALPH1 in mRNA decapping ([10] Fig. 5A). It is likely that ALPH1 orthologues of all other Kinetoplastida have predominant cytoplasmic localisations too. To investigate the localisation of an non-ALPH1 homologue, *T. brucei* ALPH2 was expressed as C-terminal eYFP fusion in procyclic trypanosomes using an inducible expression system [39]. ALPH2 showed a localisation pattern characteristic of mitochondrial proteins and co-localised with a mitochondrial stain (MitoTracker™ Orange) (Fig. 5A).

Euglena is too distantly related to the Kinetoplastida to unequivocally assign its ALPH to the ALPH1 or non ALPH1 group, but its sequence motifs (GDIHG, GDLVGKG, GNHD, HAG, VFFGH, LDTG) are more similar to the non-ALPH1 group and this is also suggested by the absence of N- and C-terminal extensions (Fig. 5A). Interestingly, Euglena ALPH is the only Kinetoplastida ALPH with a predicted trans-membrane domain. Euglena ALPH was not enriched within purified mitochondria fractions [40] and also not within chloroplasts (Martin Zoltner, Charles University in Prague, personal communication). Like Kinetoplastida, Euglena has no recognisable homologue to the canonical mRNA decapping enzyme DCP1/DCP2 in its genome and the absence of an ALPH1 homologue raises the question of how mRNA decapping is achieved in this organism.

As previously reported, Blast searches cannot identify a Dcp2 homologue in Kinetoplastida, with the one exception of Perkinsela (not examined in this study) [10]. Perkinsela is an obligate intracellular component of the Amoebozoa Neoparamoeba. Its Dcp2 has closest similarity to Dcp2 of flowering plants and the gene may have been taken up by lateral gene transfer.

### ALPHs have in vitro mRNAs decapping activity

The phylogenetic data from us and others [6] show that ApaH like phosphatases were present in the last common ancestor of eukaryotes. Since then, 60% of eukaryotes have lost the enzyme and of those ALPH proteins that are still present, 73% have predicted non-cytoplasmic localisations. ALPH proteins are pyrophosphatases and at least the related bacterial ApaH protein has a rather broad substrate specificity and cleaves for example both NpnN nucleotides [13–15] as well as mRNA capped with NpnN [24, 26]. The need to protect capped mRNAs from uncontrolled degradation may create selection for the loss or non-cytoplasmic localisation of eukaryotic ALPH proteins. To test this hypothesis, we tested ALPH proteins with non-cytoplasmic localisation prediction for mRNA decapping activity in vitro.

**Fig. 6:**
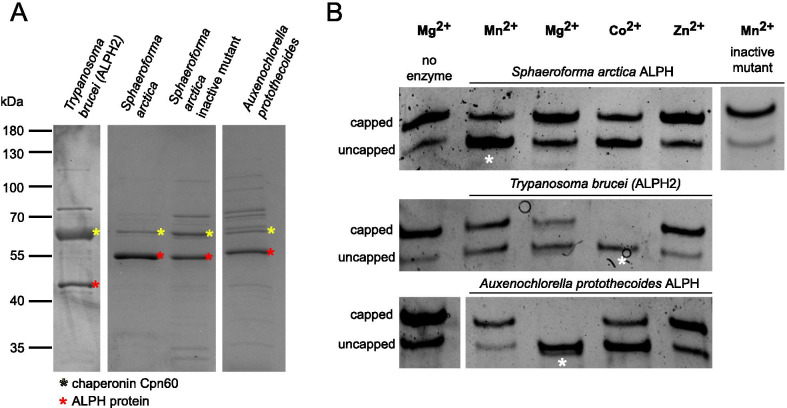
ALPH proteins have mRNA decapping activity. **A** Recombinant ALPH proteins were produced in Arctic Express DE cells and purified via Nickel affinity chromatography. **A** Coomassie stained gel loaded with the purified proteins (red asterisk) is shown. Note that the Arctic Express DE cells contain chaperons (yellow asterisk). Chaperonin Cpn60 is often co-purified, in the case of TbALPH2 to a large extent. **B** The enzymes were tested for in vitro decapping activity in the presence of 1 mM of divalent ions, as indicated. A reaction without enzyme served as negative control. For each enzyme, an asterisk marks the conditions for best decapping activity. The gels are representative gels out of at least three replicates. An inactive mutant of *Sphaeroforma arctica* has no activity (last lane)

We produced recombinant ALPH proteins from randomly selected organisms of the three eukaryotic kingdoms that have ALPHs: we used ALPH of the Ichthyosporea *Sphaeroforma arctica*, ALPH of the green algae *Auxenochlorella protothecoides* (A0A087SQ73) and ALPH2 from *Trypanosoma brucei* (ALPH2 has mitochondrial localisation and is not the mRNA decapping enzyme). As a control, ALPH of *Sphaeroforma arctica* was also produced as an inactive mutant by mutating a conserved amino acid in the metal ion binding motif (GDVIG:GNVIG [41]). All four proteins were purified from Arctic express cells (Fig. 6A) and subsequently tested in in vitro decapping assays using a 39 nucleotide long RNA oligo with a m^7^G cap structure as a substrate. Capped and uncapped oligos can be distinguished by differences in gel mobility on urea acrylamide gels complemented with acryloylaminophenyl boronic acid [42, 43]. The decapping activity of the three active enzymes was tested in the presence of different bivalent ions (Mg^2+^, Mn^2+^, Co^2+^ and Zn^2+^) (Fig. 6B). All the enzymes had mRNA decapping activity, but the ion requirements for optimal activity differed: the ALPH of *Sphaeroforma arctica* showed the highest decapping activity with Mn^2+^ and some activity with Co^2+^, but none with Mg^2+^ or Zn^2+^. *T. brucei* ALPH2 had the best decapping activity with Co^2+^ and some with Mg^2+^ and possibly Mn^2+^, but none with Zn^2+^. *Auxenochlorella protothecoides* ALPH had the best activity with Mg^2+^ and some with Co^2+^, but none with Zn^2+^ and Mn^2+^. These differences in ion requirements indicate that the enzyme activities are likely specific and not caused by contaminating bacterial enzymes, which would be expected to require identical ions in all samples. Moreover, the catalytically inactive mutant ALPH of *Sphaeroforma arctica* had no decapping activity, when tested with its supportive ion (Mn^2+^), further strong evidence for the specificity of the decapping activity.

The data show that all ALPH enzymes tested accept capped mRNA as a substrate in vitro. Importantly, the tested ALPH proteins have non-cytoplasmic localisation predictions, indicating that mRNA cannot be their physiological substrate. Instead, the findings suggest that ALPH proteins may have a similar broad substrate specificity as previously shown for bacterial ApaH. The data support the hypothesis that the preference for none or non-cytoplasmic ALPHs in many eukaryotes could serve to prevent uncontrolled mRNA degradation.

## Discussion

mRNAs are essential molecules in both eukaryotes and prokaryotes and need to be protected from unregulated exonucleolytic degradation. 5´-3´exoribonucleases require monophosphorylated RNA 5´ends and the major protection strategy is therefore to avoid such monophosphates. Most eukaryotic mRNAs are modified at their 5´end by an m^7^ methylguanosine (m^7^G) cap, linked via an inverted 5´-5´bridge [44]. A small fraction of eukaryotic RNAs carries non-canonical nucleotide metabolite caps, for example an NAD + cap [45, 46]. Bacteria were long believed to simply keep the triphosphate that is naturally present at their 5´end after transcription, but more recently were found to have diphosphate 5´ends [47], and also non-canonical metabolite caps, such as the NAD + cap [48], the 3-dephospho-coenzyme A cap (dpCoA) [49] or dinucleoside polyphosphate caps [24–26]. Archaea mRNAs are not well studied but do carry triphosphates caps [50].

In common to the increasing number of different mRNA caps and mRNA 5´ end modifications discovered throughout all kingdoms of life is the pyrophosphate bond, and therefore the requirement of a pyrophosphatase to remove the cap or the polyphosphate. Enzymes of four different families can cleave pyrophosphate bonds at the 5´end of mRNAs [51]. DXO and histidine triad (HIT) proteins, act mainly on faulty RNAs or on cap remnants or remove non-canonical NAD + caps without cleaving the pyrophosphate bond [51]. This leaves two pyrophosphatase families that act on intact mRNAs. Nudix domain proteins include the prototypic eukaryotic mRNA decapping enzyme Dcp2 [52] and several further eukaryotic nudix hydrolases with activity to the m^7^G cap and non-canonical metabolite caps [53–55] as well as the bacterial RppH [56] and NudC proteins [57] that act on di- or triphosphorylated mRNAs and on NAD + caps [51]. Enzymes of the fourth group, the ApaH family only entered the field of mRNA decapping most recently. The ApaH like phosphatase ALPH1 from *Trypanosoma brucei* was shown to be the only or major mRNA decapping enzyme in these parasites [10]. Bacterial ApaH cleaves of non-canonical nucleoside-tetraphosphate caps [24–26] and likely regulates gene expression during stress.

The major aim of this work was to find out how widespread mRNA decapping by ApaH like phosphatases is. To identify ALPH proteins in all available eukaryotic proteomes, we have developed a Python based algorithm based on recognising the six conserved motifs of ALPH. We detected 441 ALPH proteins in 332 of 827 organisms. The full details of these 441 ALPH proteins are summarized in Additional file 3: Table S1 and contribute to a more comprehensive knowledge of this enzyme family.

**Fig. 7:**
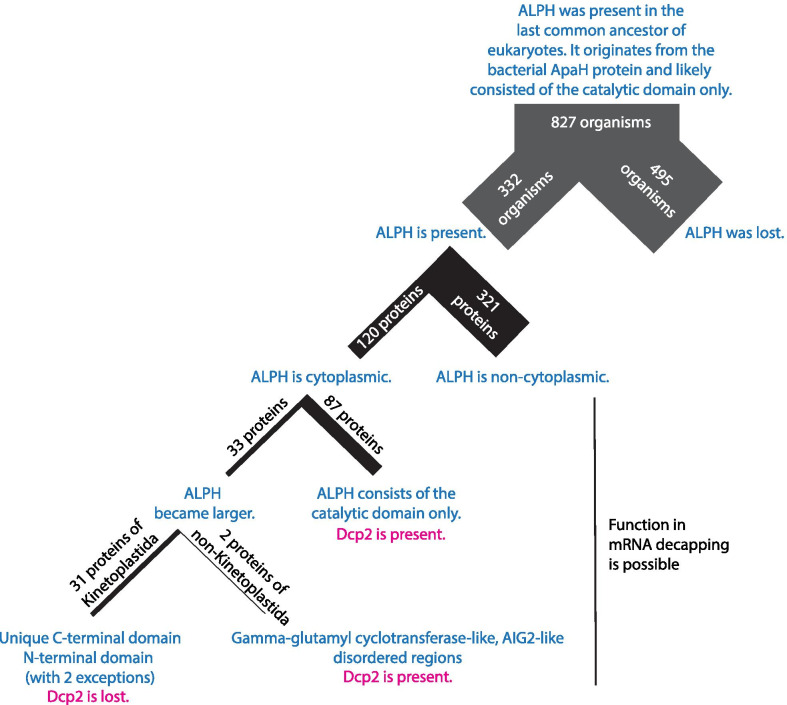
Summary of the major findings of this work. For the ALPH proteins of Kinetolastida, non-cytoplasmic localisation was assumed for the non-ALPH1 orthologues and cytoplasmic localisation for ALPH1 orthologues, based on localisation studies in *Trypanosoma brucei* (Fig. 5)

What is the likelihood for these 441 ALPH proteins to function in mRNA decapping? The most stringent criterion is intracellular localisation: mRNA decapping enzymes must be cytoplasmic to access their substrates. 321/441 ALPH proteins have non-cytoplasmic localisation prediction and these predictions correlate in many cases with predicted transmembrane helices and signal peptides (Fig. 7). For two ALPH proteins, yeast Ppn2 and *T. brucei* ALPH2, non-cytoplasmic predictions were confirmed experimentally [7, 37, 38], this work). This leaves 120 ALPH proteins with predicted cytoplasmic localisation. A second essential feature of an mRNA decapping enzyme is the need to regulate its activity: For the nudix domain protein Dcp2, this regulation is done by the N-terminal domain, which mediates interactions with decapping enhancers [58]. The *T. brucei* mRNA decapping enzyme ALPH1 has unique N- and C-terminal extensions and the C-terminal extensions are conserved across all Kinetoplastida ALPH1 homologues and it is therefore tempting to speculate that they contribute to enzyme regulation. 87 of the 120 ALPH proteins with cytoplasmic predictions consist of the catalytic domain only (Fig. 7). The absence of additional sequences does not preclude the protein from being an mRNA decapping enzyme, but any interaction with putative decapping regulators must occur directly via the catalytic domain. The remaining 33 proteins that have sequences in addition to the catalytic domain include the 31 Kinetoplastida homologues to the *T. brucei* mRNA decapping enzyme ALPH1, the ALPH protein of the fungi *Moniliophthora roreri* (Gamma-glutamyl cyclotransferase-like domain) and the ALPH protein of *Ectocarpus siliculosus* (disordered regions) (Fig. 7). A third criteria to evaluate the likelihood of an ALPH enzyme to act in decapping is to look for the presence of an alternative mRNA decapping enzyme. Orthologues to the mRNA decapping enzyme Dcp2 were readily detectable by Blast in all organisms with cytoplasmic ALPH proteins, with the marked exception of the Kinetoplastida. To summarize, if any ALPH proteins from non-Kinetoplastida function in mRNA decapping, they are unlikely the exclusive mRNA decapping enzyme and their regulation will, with two possible exceptions, entirely depend on the catalytic domain.

All three ALPH proteins that we tested had mRNA decapping activity in vitro, even though none of these enzymes is likely to use mRNA as their physiological substrate as all three have (predicted or proven) mitochondrial localisation. This broad substrate range of ALPHs is not unexpected: the related bacterial ApaH protein cleaves NpnN nucleotides [13–15] as well was mRNA capped with NpnN [24, 26] and even has phosphatase and ATPase activity in addition to its pyrophosphatase activity [59]. The only other characterised ALPH protein, the *S. cerevisiae* ALPH protein Ppn2 has, to our knowledge, not been tested for mRNA decapping activity. Substrate unspecificity is not restricted to ApaH and ApaH like phosphatases, but also found in (decapping) enzymes of the nudix domain family [60]: the major bacteria decapping enzyme RppH was initially identified as diadenosine polyphosphate hydrolase [61] and can also cleave NAD^+^ caps [55]. Recent in vitro studies of mammalian nudix proteins shows that many have mRNA decapping activity towards a range of caps and some also towards free metabolites [53, 55, 62, 63]; how many of these nudix proteins have mRNAs as their natural substrates is still debated.

The broad substrate range of ALPHs that includes mRNA caps provides an explanation for its loss or non-cytoplasmic localisation in most eukaryotes. The last common ancestor of all eukaryotes had ALPH. This assumption is supported by the presence of ALPH proteins in all eukaryotic kingdoms found in this and earlier [6] studies. With the evolution of the eukaryotic mRNA cap, eukaryotes faced two problems: first, the cap needed to be protected from unregulated degradation by pyrophosphatases and second, an mRNA decapping enzyme was needed for regulated degradation. The first challenge was likely addressed in multiple ways, including for example protection by cap binding proteins like the eIF4F complex. One important evolutionary strategy may have been to inactivate the bacterial-inherited ALPH proteins. Many eukaryotes fully lost these bacterial-derived enzymes or at least did not tolerate cytoplasmic localisation. Evolution has solved the second challenge by evolving pyrophosphatases as regulatable mRNA decapping enzymes. Usually, nudix hydrolases were exploited for this task, but Kinetoplastida uniquely use one of their ALPH proteins. Some non-Kinetoplastida have ALPH proteins with predicted cytoplasmic localisation: if these ALPH proteins are indeed cytoplasmic, they could act in mRNA decapping in addition to Dcp2 or else, they may have been evolved to not accept capped mRNA as a substrate.

One open question is whether all organisms outside the Kinetoplastida use DCP2 orthologues as their major mRNA decapping enzyme. While DCP2 has been functionally characterised in yeast [52], human [64] and plants [65], there are no experimental data on Metamonada and Discoba. Metamonada species possibly have a DCP2 homologue: in *Trichomonas* this can be identified by Blast and in *Giardia* by more sophisticated in silico methods [66, 67]. Whether Discoba have DCP2 is unclear. A possible DCP2 homologue is identifiable by blast in the Discoba *Naegleria*, but not in Euglena. The question whether DCP2 was present in the last common ancestor and selectively lost in Kinetoplastida, or whether it evolved subsequently to the separation of the Discoba cannot be answered without experimental data.

In the last few years, more and more mRNA cap variants have been discovered in both prokaryotes and eukaryotes, and more and more enzymes that act in mRNA decapping, including ApaH like phosphatases and bacterial ApaH. Our analysis of eukaryotic ApaH like phosphatases is an important contribution towards a more comprehensive picture of mRNA decapping.

## Conclusions

Despite being present in all eukaryotic kingdoms, ApaH like phosphatases are poorly understood with only a few studies available. Here, we present a new algorithm for the identification of ALPH proteins and we provide a comprehensive dataset of ApaH like phosphatases of all available reference proteomes. Trypanosomes and possibly a few other organisms use ALPH proteins for mRNA decapping; however, the physiological functions of the vast majority of eukaryotic ALPH proteins remain to be discovered. In vivo experiments are essential, as the wide substrate range of ALPH will impede the identification of physiological substrates in vitro.

## Methods

### Identification of ApaH like phosphatases

A novel algorithm was developed using Python 3 to identify ALPHs in a set of proteome fasta files. The algorithm initially used narrow matrices to identify the 6 conserved motifs (4 common to all PPP and 5–6 unique to ALPHs) based on the data of [3, 6], these matrices were successively extended manually using a set of yeast proteomes and euglenozoan proteomes as training sets, controlled by BLAST. These initial screens gave information about conserved and non-conserved distances between the 6 motifs. Proteins that had only one motif missing were manually inspected further. If they possessed the missing motif in an only slightly different version (e.g. only one amino acid altered in a conservative way) and, at the same time, had the correct distances between the motifs (where these distances are conserved), the motif matrix was extended to include this motif. One exception was made for R at position 6 in motif 2: this is not tolerated, as it prevented the clear distinction of ALPHs from the related ApaH and RLPH enzymes [6]. Several rounds of manual iterations and matrix improvements resulted in an algorithm that uses one narrow matrix for the initial screen and, if not all motifs were found, the algorithm tolerates up to two motifs from an extended matrix. Furthermore, we included restrictions to three of the distances between the motifs. This algorithm was run with all 827 proteomes and identified 430 ALPH proteins. Thereafter, we screened all 827 proteomes for ‘one motif missing’, successively at each position (data shown in Additional file 4: Table S2a). Manual analysis of these data resulted in three further changes to the matrix and identification of 11 additional ALPH proteins (details in Additional file 4: Table S2a). The final software was run again on all 827 proteomes but did not identify any additional ALPH proteins. In the final version of the software, the narrow matrices are defined as [G, D, VILT, HQ, G], [G, DN, LMIFVT, EIVTLAC, NSATFGVQIDHM, KQN, GASHT], [G, N, HNWQ, ED], [H, AG, G], [VIT, IVYFLAMT, FY, G, H], [LVIMT, DE, STG, GASRN] for motif 1, 2, 3, 4, 5 and 6, respectively. The extended matrices are defined as [GxX, DxX, VILTxX, HQxX, GxX], [GxX, DNxX, TIFLYVMxX, ETIALCVMxX, EQTIASDFGHCVNMxX, KQNxX, GASHTxX], [GxX, NxX, HNWQxX, EDxX], [HxX, AGLVxX, GxX], [AVILCMTxX, IVYFLAMTxX, FYxX, GxX, HxX], [LVIMTxX, DExX, LATGSVxX, LMGRASNExX] for motif 1, 2, 3, 4, 5 and 6, respectively. Furthermore, the algorithm restricts the distances between the motifs to 14–150 (motif 1 to 2), 25–80 (motif 2 and 3) and 17–40 (motif 5 to 6). As one further control to ensure that all ALPH proteins were identified, we screened all 827 proteomes again with matrices tolerating more amino acids at positions in the motifs that appeared less conserved (matrices shown in Additional file 4: Table S2b): again, no further ALPH proteins were identified.

ALPHs with missing motifs due to wrongly annotated start codons or sequencing errors will not be detected, with the exception of an unidentified amino acid included in the extended matrix (x, X). For the Kinetoplastida, such ALPHs were manually included based on the available genome information from TriTrypDB [28, 29]; for all other organisms, such truncated ALPH proteins (11 in total) are listed in Additional file 4: Table S2c.

Finally, all proteins were manually inspected: even though the extended sequence matrix would allow the presence of motifs with little homology to the consensus motif, we found that the extended matrix was used rather rarely (1.9% of all motifs, affecting 11.3% of all ALPH protein; no ALPH protein had 2 motifs form the extended matrix) and if so, the extended motifs had still most amino acids from the narrow matrix. The Python programme is available in Additional file 2: Python Software.

### Cloning and expression of recombinant proteins

All ALPH proteins were expressed either full length (*T. brucei* ALPH2 = Tb927.4.4330) or with minor N-terminal truncations to exclude the transmembrane regions (*Sphaeroforma arctica*: amino acids 21–316; *Auxenochlorella protothecoides*: amino acids 21–327) in Arctic Express DE competent cells (Agilent). At the N-terminus, the proteins were fused to a tag consisting of a 6xHis-Tag, a Thrombin cleavage site, a SUMO tag and a TEV cleavage site, resulting in the following additional protein sequence:

MGSSHHHHHHSSGLVPRGSASMSDSEVNQEAKPEVKPEVKPETHINLKVSDGSSEIFFKIKKTTPLRRLMEAFAKRQGKEMDSLRFLYDGIRIQADQTPEDLDMEDNDIIEAHREQIGGHMENLYFQGEASAT. For cloning reasons, all proteins had the extension GSGSGC at the C-terminus. For the inactive mutant of the *Sphaeroforma arctica* ALPH, the highly conserved GDVIG motif involved in metal ion binding was mutated to GNVIG [41]. The purification of the recombinant proteins was done via Nickel-agarose beads using standard procedures as previously described [10], except that expression in Arctic Express DE cells was done at 10 °C, following the instructions of the company. The concentration of all purified proteins was estimated from Coomassie gels using a titration of a protein with known concentration for calibration.

### Phylogeny

The tree of the Euglenozoa ALPHs (Fig. 5) was done with all ALPH catalytic domains (defined as starting 6 amino acids upstream of motif 1 and ending 8 amino acids downstream of motif 6) using MEGA X [68, 69]. All the sequences were aligned using Muscle (gap open −2.90; gap extend: 0.00; Hydrophobicity Multiplier: 1.20; Max Iterations: 16; Cluster Method: UPGMA; Min Diag Length (Lambda): 24) [70] and gaps were manually removed. The best protein substitution model was calculated: JTT + G + I (BIC = 23908). The tree was built using the Maximum Likelihood method (500 bootstrap cycles) and the JTT matrix-based model [71]. The tree with the highest log likelihood (−11295.89) is shown. The percentage of trees in which the associated taxa clustered together is shown next to the branches. Initial tree(s) for the heuristic search were obtained automatically by applying Neighbor-Join and BioNJ algorithms to a matrix of pairwise distances estimated using the JTT model, and then selecting the topology with superior log likelihood value. A discrete Gamma distribution was used to model evolutionary rate differences among sites (5 categories (+ G, parameter = 1.0217). The rate variation model allowed for some sites to be evolutionary invariable (+ I, 6.38% of sites).

Different methods (minimal evolution, neighbour joining) gave slightly different trees, but, importantly, with all methods, the ALPH1 isoforms group together in one clade that never contains non-ALPH1 isoforms.

## Proteomes

Proteomes were downloaded from Uniprot, using all available reference proteomes of UniProt_release 2019_02 (For Archaea: 2019_11). [27]. Additional file 3: Table S1a lists all proteomes used in this work. For Kinetoplastida, we downloaded all available proteomes from TriTrypDB (February 2019) [28, 29]. During the revision process, we screened UniProt_release 2021_01 selectively for all phylogenetic groups (Fig. 1) that had no reference proteomes available in UniProt_release 2019_02: this search identified proteomes of two Haptista and one Dinoflagellate.

### Work with trypanosomes

*Trypanosoma brucei* Lister 427 procyclic cells expressing a TET repressor (pSPR2.1) were used for all experiments [39]. Cells were cultured in SDM-79 [72] at 27 ℃ and 5% CO_2_. Transgenic trypanosomes were generated using standard procedures [73]. All experiments used logarithmically growing trypanosomes. The mitochondria were stained by adding 1 µM MitoTracker™ Orange CMTMRos (Invitrogen) to living cells; imaging was done within 30 min.

### Microscopy

Z-stack images (100 stacks at 100 nm distance) were taken with a custom build TILL Photonics iMic microscope equipped with a sensicam camera (PCO), deconvolved using Huygens Essential software (Scientific Volume Imaging B. V., Hilversum, The Netherlands). All images are presented as Z-projections (method sum sliced) produced by ImageJ software.

### In vitro decapping assays

Each decapping reaction was done in 10 µl volume (with RNAse-free water) containing 50 mM Tris–HCl (pH 7.9), 100 mM NaCl, 1 mM metal ions (variable), 40 units Ribolock RNAse inhibitor (ThermoFisher Scientific), 0.25 µM recombinantly-produced ALPH enzyme and 0.39 µM capped RNA oligo m^7^G-AACUAACGCUAUUAUUAGAACAGUUUCUGUACUAUAUUG (Bio-Synthesis, Texas, USA). The sample was incubated for 1 h at 37 °C. Control reactions were done without enzymes. The reaction was stopped by ethanol precipitation: 1 µl 3 M RNAse-free sodium acetate (pH 5.5) (Ambion), 1 µl RNAse-free glycogen (ThermoFisher Scientific) and 30 µl cold 100% ethanol was added, the sample incubated at −20 °C for 30 min, followed by centrifugation (15 min, 20,000 g). The pellet (containing the RNA oligo) was washed once in 80% ethanol, air-dried, resolved in 5 µl RNAse-free water (ThermoFisher Scientific) and either stored at −80 °C or directly prepared for loading to the gel.

RNA Samples were separated on urea acrylamide gels that contained acryloylaminophenyl boronic acid [42, 43]: boronyl groups form stable complexes with the m^7^G cap structure, this way increasing the difference in gel mobility between capped and uncapped oligos [42, 43]. For 12% gels, a 10 ml gel solution was freshly prepared with distilled water, containing 12% acrylamide/bis-acrylamide (19:1) (Sigma-Aldrich), 7 M urea (Sigma-Aldrich), 0.1% ammonium persulfate, 4 µl TEMED, 0.25% acryloylaminophenyl boronic acid (Sigma-Aldrich) and 100 mM Tris–acetate (pH 9.0). Gels were run in 0.5 × TBE buffer (45 mM Tris–HCl pH 8.3, 45 mM boric acid, 1 mM EDTA) using the Mini-PROTEAN Tetra Vertical Electrophoresis cell (gel size 10 × 8 cm, BioRad). Each run was preceded by a 30 min pre-run at 300 V to warm the gel. The purified RNA samples were prepared for gel electrophoresis by adding 1 µl loading dye (gel loading buffer II, Invitrogen) followed by incubation at 95 °C for 5 min. Samples were loaded immediately to the pre-rinsed wells of the pre-run gel and the gel was run at 200 V (after a 10 min run at 170 V) for 30–40 min, stained for 20 min in SYBR™ Green II RNA Gel Stain (1:10,000 in 0.5 × TBE, ThermoFisher Scientific) and imaged on the iBright™ (Invitrogen) with nucleic acid settings.

## Supplementary Information

**Additional file 1:** **Figure S1**. Phylogenetic tree of ALPH in eukaryotes.

**Additional file 2:** Python Software.

**Additional file 3: Table S1.** List of all organisms that were screened for the presence of ALPH  and detailed information on all ALPH proteins that were identified in this study.

**Additional file 4: Table S2.** a) Documentation of the last step of software improvement: the ‘missing motif screen’ on the complete set of proteomes. b) extended control matrix. c) list of ALPH proteins with truncated N- or C-termini.

**Additional file 5: Table S3.** The 285 available reference proteomes for Archaea were downloaded from UniProt on 7th of January 2020. They were screened with the Python programme for the presence of ALPH. Only one proteome contained one ALPH.

## Data Availability

All data generated or analysed during this study are included in this published article and its Additional files.
