# Supplementary material for: Is mRNA decapping by ApaH like phosphatases present in eukaryotes beyond the Kinetoplastida?
Source: BMC Ecol Evol. 2021 Jun 23;21:131. doi: 10.1186/s12862-021-01858-x (PMC8220851; doi:10.1186/s12862-021-01858-x)
Supplement: Supplementary file 1 — Additional file 1: Figure S1. Phylogenetic tree of ALPH in eukaryotes. [file 12862_2021_1858_MOESM1_ESM.pdf]

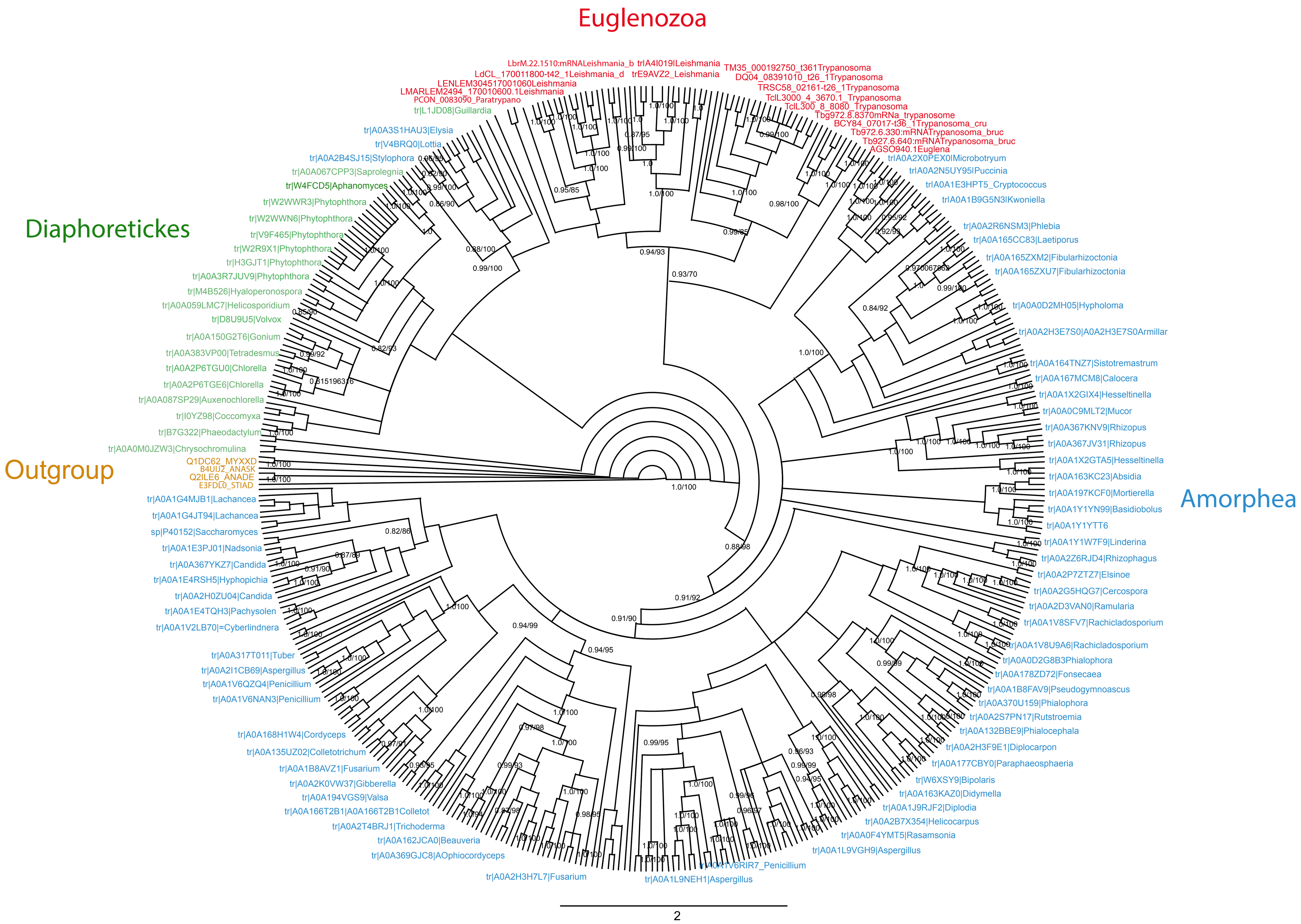

Figure S1: Posterior probability and Maximum Likelihood tree of ALPH in eukaryotes.
This tree was built from catalytic domains (insertions removed) of all eukaryotic ALPH proteins; all sequences are listed in Supplementary Table S1.The Mr Bayes (posterior probability) and PhyML (maximum likelihood) trees were inferred using substitution models determined by Prot-Test (1). Boot- strapping was performed for 1000 replicates and the best tree topology was inferred. Bootstrap values above 80 are significant for PhyML and 0.95 for Mr Bayes.
Outgroup - Myxococcales species
.

(1) Abascal, F., Zardoya, R. & Posada, D. ProtTest: selection of best-fit models of protein evolution. Bioinformatics 21, 2104–2105 (2005).
